# Microleakage of Three Types of Glass Ionomer Cement Restorations: Effect of CPP-ACP Paste Tooth Pretreatment

**Published:** 2015-09

**Authors:** Maryam Doozandeh, Fereshteh Shafiei, Mostafa Alavi

**Affiliations:** 1 Dept. of Operative Dentistry, School of Dentistry, Shiraz University of Medical Sciences, Shiraz, Iran.; 2 Biomaterial Research Center, Dept. of Operative Dentistry, School of Dentistry, Shiraz University of Medical Sciences, Shiraz, Iran.; 3 Undergraduate Student, School of Dentistry, Shiraz University of Medical Sciences, Shiraz, Iran.

**Keywords:** Microleakage, Glass Ionomer Cement, Case in Phosphopeptide- Amorphous Calcium Phosphate

## Abstract

**Statement of the Problem:**

Casein phosphopeptide–amorphous calcium phosphate (CPP-ACP) increases the mineral content of tooth structure. This may enhance the chemical bonding of glass ionomer cements (GIC) and marginal sealing of their restorations.

**Purpose:**

The aim of this study was to evaluate the effect of CPP-ACP paste pretreatment on the microleakage of three types of GIC.

**Materials and Method:**

In this study, 72 Class V cavities were prepared on the buccal and lingual surfaces of molars with occlusal margins in enamel and gingival margins in root. The cavities were divided into 6 groups. Cavities in group 1 and 2 were restored with Fuji II, group 3 and 4 with Fuji II LC, and group 5 and 6 with Ketac N100 with respect to the manufacturers’ instructions. In groups 2, 4 and 6, CPP-ACP containing paste (MI paste) was placed into the cavities for 3 minutes before being filled with GIC. The teeth were thermocycled, stained with dye, sectioned, and scored for microleakage under stereomicroscope. Kruskall-Wallis and Chi-Square tests were used to analyze the data.

**Result:**

There were no statistically significant differences between the control and the CPP-ACP pretreatment groups in enamel and dentin margins. In pairwise comparisons, there were no significant differences between the control and the experimental groups in enamel margin, and in dentin margins of G1 and 2, G5 and 6; however, a significant differences was detected in dentin margins between G3 and 4 (*p*= 0.041).

**Conclusion:**

CPP-ACP paste pretreatment did not affect the microleakage of Fuji II and Ketac N100 in enamel or dentin, but decreased the microleakage in dentine margins of Fuji II LC when cavity conditioner was applied before surface treatment.

## Introduction

Durable bond between tooth and restorative materials is crucial in long-term success of restorations in dentistry.[1] Different varieties of restorative materials are used to restore carious or non-carious lesions of teeth. Glass ionomer cements (GICs) were introduced by Wilson and Kent; since then, these materials have been applied in wide clinical uses such as liner, base, restorative materials and luting agent.[2] GICs are water-based tooth-colored restorative materials which consist of polyalkenoic acid and a glass component in an acid-based reaction setting.[3] Conventional GICs have the drawbacks like handling and working difficulties, sensitivity to water, and brittleness.[4] These shortcomings were improved by adding photosensitive methacrylate resin to glass powder in resin-modified glass-ionomer cements (RMGIC). This addition results in increased physical and mechanical bonding strength to tooth structures.[5] A novel nano-filled RMGIC, named nano ionomer (NI), was presented with superior mechanical properties compared with conventional RMGIC.[6]

GICs are the only self-adhesive dental materials in clinical applications.[7] A chemical reaction between the carboxylic groups of polyalkenoic acid with calcium ions of hydroxyapatite (HAP) at tooth interfaces results in ionic bond between GIC and dental hard tissues.[8] In RMGIC, adhesion to tooth structure might be improved by the formation of hybrid layer from the hydrophilic methacrylate resins besides an ionic bonding like GIC. Application of cavity conditioner before restoration plays an important role in developing effective bond with RMGIC.[9] Pretreatment with polyalkenoic acid could remove the smear layer, partially demineralize dentin, and improve the chemical and micromechanical bonding of RMGIC.[10]

Despite the evolution of different types of GIC, several studies demonstrated that none of these materials have a complete marginal sealing, which is considered as a major reason for microleakage.[11-12]

Casein phosphate amorphous calcium phosphate (CPP-ACP) is used in several commercial products like chewing gum and mouth rinses. It is one of the treating methods for reduction of tooth hypersensitivity in dental applications.[13] Stabilization of calcium phosphate by casein phosphopeptide creates highly concentrated calcium phosphate ions on tooth surfaces. This can remineralize the enamel and dentin subsurface and enhance their resistance to subsequent demineralization.[13-15] Fluoride intervention, alone or in combination with CPP-ACP, is an effective method in decreasing the incidence and progression of non-carious cervical lesions.[16]

Application of CPP-ACP pretreatment was assumed to provide a reactive substrate by precipitation of calcium phosphate.[15] Adebayo *et al.* found that dentin preconditioning with CPP-ACP paste (for 60 min per day and repeated for 7 days) did not have any adverse effect on the resin-dentin interfacial morphology[17] and microshear bond strength (µSBS) of Clearfil SE Bond; though it decreased the µSBS of G Bond.[18] In another study, Borges *et al. *reported that the increased calcium availability after dentin pretreatment with a CPP-ACP (MI paste) for 3 min could enhance the bond strength of Adper SE Plus, but could not influence the adhesion of Clearfil SE Bond.[19] Mazzaoui *et al.* [20] and Prabhakar *et al.* [21] demonstrated that the incorporation of CPP-ACP into GIC could enhance the remineralization and protect the dentin surfaces during acid challenge. Since CPP-ACP produces a highly gradient concentration of mineral content in dental hard tissue, it is assumed that this pretreatment (for 3 min) could increase the chemical bond of GIC and their subsequent seal. To the best of authors’ knowledge, there is no study concerning the effect of this application procedure on the marginal sealing of different types of GIC to tooth surfaces. Therefore, the aim of this study was to compare the effect of CPP-ACP surface pretreatment on microleakage of conventional GI, RMGI and NI. The null hypothesis was that CPP-ACP pretreatment would have no effect on the marginal sealing and adaptation of three types of GICs compared with no-treatment groups.

## Materials and Method

Thirty-six intact human molars were employed in this study. The teeth were cleaned with dental prophylactic cups and low speed handpiece, rinsed, and stored in %1 chloramine-T solution for one week. They were then immersed in distilled water at 4˚C before being used in this study.

Seventy-two standardized Class V cavities (3mm width, 2.5mm depth, 2mm height) were prepared on the buccal and lingual surfaces of teeth in a way that occlusal margins were placed in enamel and gingival margins in dentin, 1mm under cemento-enamel junction. Straight fissure burs (# 835/010; Teeskavan, Iran) were used to prepare cavities with a high speed handpiece in presence of air-water spray. The burs were replaced after every five preparations. Dimensions of all cavities were checked with a periodontal probe. The cavities were rinsed and dried. Then, they were randomly assigned into six groups (n=12). The restorative procedures were done as follows. In group1, the liquid and powder of self-cured GI (Fuji II; GC. Tokyo, Japan) in a 2.7/1 ratio (1 level scoop of powder to 1 drop of liquid, according to the manufacturer’s instruction) was mixed and placed in cavities in one increment. The contour was formed and a matrix was placed until the end of setting. In group 2, the cavities were first actively pretreated with a CPP-ACP product, MI paste (GC; Tokyo, Japan) for 3 minutes by using a brush. The excess was removed with an absorbent paper while the tooth surfaces were still kept moist.[19] Then, the self-cured GI was placed into cavity similar to group 1.In group 3, the cavity conditioner (GC; Tokyo, Japan) was applied into cavities for 10 s. After rinsing and drying, RMGIC (Fuji II LC, GC, Tokyo, Japan) in 2.3/1 ratio by weight (1 level scoop of powder to 1 drop of liquid, according to manufacturer’s instruction), was mixed and used for restoring the cavities. After contouring, according to manufacturer’s instructions, the restorations were light cured for 40s with a halogen unit (600mW/cm[2]; VIP Junior, BISCO, Shaumburg, IL, USA).In group 4, after applying the cavity conditioner like previous group, MI paste was placed into cavities as mentioned above. Then RMGIC was used for restoring teeth and light-curing was performed as in group 3.In group 5, nano primer of Ketac N100 (3M ESPE, Seefeld, Germany) was brushed into cavities for 15s. After air drying (shiny surface), the primer was cured for 10s. The base and catalyst of NI (Ketac N100, 3M ESPE) was mixed and placed into cavities in one increment and got light-cured for 30s.Finally, in group 6, MI paste was first used to pretreat the cavities (similar to group 2) and then restoration of the teeth was done as described in group 5.

All preparations and restorations were performed by the same operator at room temperature. After 24 hours of water storage at 37˚C, the restorations were finished with aluminum oxide discs (Sof- Lex; 3M ESPE, USA). The teeth were kept in water for 1 week and then thermocycled at 5˚C and 55˚C with a 30-second dwell time (1000 cycles).[11]

The root apices and furcation areas were sealed with utility wax. Two layers of nail varnish were used to coat all surfaces of teeth except for the restoration and 1mm beyond the margins. The teeth were immersed in 1% methylene blue solution for 24 hours. After being rinsed and dried, they were sectioned longitudinally through the center of restorations in a buccolingual direction with a water-cooled diamond saw (Leitz 1600; Wetzlar, Germany). Two independent evaluators examined the tooth sections for dye penetration by using a stereomicroscope (Carl Ziess Inc; Oberkochen, Germany) at 20× magnification.

**Figure 1:**
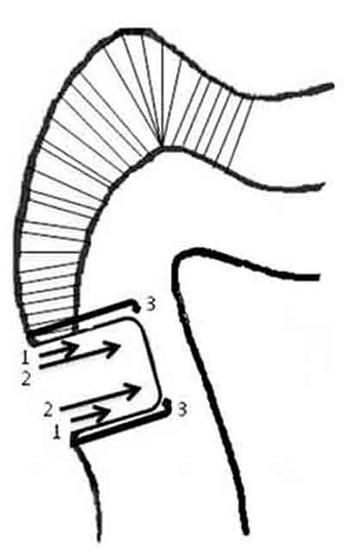
Schematic picture of microleakage score used for analyzing dye penetration

Non parametric scale from 0 to 3 was used to analyze the dye penetration as follows: 0=no dye penetration, 1=dye penetration less than ½ of the cavity depth, 2=dye penetration more than ½ of the cavity depth, 3=dye penetration through the axial wall (Figure 1).[11-12] Kruskal-Wallis and Chi-square tests were adopted to analyze the results (α=0.05).

## Results

The microleakage score, median and standard deviation of dye penetration in enamel and dentin are summarized in Table 1. The Kruskal-Wallis test showed no significant differences between the control and CPP-ACP pretreatment groups either in enamel (*p*= 0.387) or in dentin (*p*= 0.092). Chi-square test was performed for pairwise comparison of microleakage within each group (Table 2). There were not any significant differences between control groups and CPP-ACP surface treatment groups in enamel margin, and in dentin margins of groups 1 and 2, and groups 5 and 6 (*p*> 0.05). However, this test revealed a significant difference in dentin margins between groups 3 and 4 (*p*= 0.041).

**Table 1:** The microleakage score and median (SD) of dye penetration in enamel and dentin

**Score ** **Groups **	**Enamel**					**Dentin**				
	**0**	**1**	**2**	**3**	**Median(SD)**	**0**	**1**	**2**	**3**	**Median(SD)**
1) Self-cured Glass-ionomer (GI)	6	3	1	2	0.5(1.16)	2	4	2	4	1.5(1.15)
2)CPP+GI	7	2	2	1	0.0(1.05)	6	4	1	1	0.5(0.96)
3)Resin Modified Glass-ionomer (RMGI)	5	4	2	1	1.0(099)	3	5	2	2	1 (1095)
4)CPP+RMGI	5	4	3	0	1.0(0.83)	8	2	1	0	0.0(67)
5)Nano-ionomer(NI)	2	4	3	3	1.5(1.08)	7	2	1	2	0.0(1.19)
6)CPP+NI	4	5	3	0	1.0(0.79)	6	3	2	1	0.5(1.02)
*P*-value	0.387					0.091				

**Table 2:** Frequency of occurrence of microleakage in enamel and dentin margins

**Groups**	**Enamel**	**Dentin**
	**Occurrence of leakage**	
1) Self-cured Glass-ionomer (GI)	6 (50%)	10 (83.3%)
2) CPP+GI	5 (41.7%)	6 (50%)
3)Resin Modified Glass-ionomer (RMGI)	7 (58.3%)	9 (75%)
4) CPP+RMGI	7 (58.3%)	4 (33.3%)
5) Nano-ionomer(NI)	10 (83.03%)	5 (41.7%)
6) CPP+NI	8(66.7%)	6(50%)

## Discussion

Adequate enamel and dentin marginal sealing of restorative material is necessary for successful restoration of tooth. The presence of chemical bonding on tooth structure plays an important role in marginal integrity, stability, and extending the longevity of adhesive bond.[22] In the current study, the microleakage of three types of glass ionomer restoration placed in Class V cavities was examined using dye penetration test. Microleakage is an important feature in evaluating the success of any restorative material used in tooth restorations.[23]

In this study, no statistically significant difference was observed between the group treated with CPP-ACP and control groups in self-cured GICs, nanofilled RMGI in enamel and dentin margins, and in enamel margins of RMGI but a significant reduction in dentin marginal leakage was found for the RMGI restoration. Therefore, the null hypothesis was partly rejected.

As previously stated, CPP-ACP has caries-preventive effect because of buffering capacity and increasing the mineral content and decreasing the demineralization of tooth.[13, 15, 20] The cariostatic effect of GIC is attributed to the fluoride release in a long period of time.[24-25] The reinforcing effect of fluoride on calcium phosphate precipitation (fluorapatite) over the tooth surfaces can increase the remineralization.[26] However, sufficient level of calcium phosphate ions must be present for effective remineralization.[27]

Pretreatment of tooth structure with MI paste before glass ionomer application may produce extra level of ions for this process. To increase the mineral content of tooth structure, the tooth surfaces were actively treated with MI paste for 3 minutes immediately after cavity preparation; which was similar to the method used in the study of Borges *et al.* This can be applicable for dentists before adhesive procedures.[19]

Mazzaoui *et al.* demonstrated that the release of fluoride and CPP-ACP from GI containing CPP-ACP protected the adjacent dentin subsequent acid challenge.[20]

In the study by Adebayo *et al.*, tooth mousses did not significantly affect the µSBS of Clearfil SE Bond or G Bond in the presence of smear layer.[18] Also, the tooth mousses did not adversely affect the resin-dentin interfacial morphology.[17] The beneficial effect of initial mild dentin demineralization was found to enhance the remineralization followed by the application of CPP-ACP.[15]

In this study, no conditioner was used before self-cured GICs. Several studies demonstrated that cavity conditioner had no effect on the bond strength and microleakage of self-cured GICs.[28-29] We speculated that the presence of smear layer may interfere with the infiltration of paste to underlying dental surfaces and reduce the subsequent effect. Tani *et al.* showed that the coarser and thicker smear layer was seen followed by the application of CPP-ACP paste on the dentin prepared under the standard condition.[30]

In our study, cavity conditioner was applied before using CPP-ACP paste in the RMGI as recommended by the manufacturer and significant reduction in microleakage was found in dentinal margins after paste pretreatment, with no effect in enamel margins.

The polyacrylic acid conditioner partially removes the smear layer without complete removal of calcium ions available for chemical bonding and remineralization by CPP-ACP.[31-32] It has the capacity to bind with the hydroxyapatite and collagen molecules so that a polymeric layer can be formed on conditioned enamel and dentin after rinsing.[33-34]

It was assumed that the demineralized conditioned dentin surfaces had less mineral content for chemical bonding with the carboxylic groups in the RMGI. After the application of CPP-ACP paste on these surfaces, the increased level of available calcium and phosphate ions might have improved the bond between RMGI and treated dentin surface.[15] This could be reflected in significant reduction of microleakage in paste treated dentinal margins. On the other hand, ES-Souni *et al.* showed that the previously mentioned polymeric layer on conditioned enamel surfaces would increase the bond strength of GICs.[31] Therefore, increased deposition of calcium phosphate ions on this polymeric layer after CPP-ACP treatment had no effect on subsequent bonding of RMGI. Therefore, no difference was found in enamel marginal sealing after paste pretreatment.

Ketac N 100 was the other RMGI used in this study. Although we removed smear layer in Ketac N 100 RMGI by its primer like the conventional RMGI in group 2, it did not improve the sealing ability of enamel and dentin after surface treatment. Similarly, Borges *et al.* demonstrated that dentin pretreatment with MI paste had no effect on bond strength of the two-step mild self-etched adhesive systems (Clearfil SE bond and Filtek P90 adhesive); however, it increased the bond strength of Adper SE Plus by increasing the availability of calcium phosphate in dentin surfaces.[19]

Ketac primer (conditioner for Ketac nano) is a mild acidic primer (pH=3) that partially removes the smear layer, improves the wettability of tooth, and increases the monomer penetration into the underlying surface. It also contains unsaturated carbon-carbon bonds that may lead to direct covalent bonding with the resin of Ketac nano after polymerization.[35]

Therefore, the primary bonding mechanism of Ketac nano can be similar to other mild self-etched resin adhesives; and the chemical interaction between the polyalkenoic acid and calcium in hydroxyapatite is considered as a secondary bonding mechanism.[6, 36] According to the results of this study, it may be assumed that increasing the mineral content of tooth surfaces by CPP-ACP had no effect on marginal sealing of NI.

Since the chemical bonding with calcium in tooth surface takes place in GICs, enamel margin in all groups revealed better sealing than gingival margins in the current study.

To the best of authors’ knowledge, there was no published study evaluating the effectiveness of CPP-ACP paste pretreatment during glass ionomer application. So, further chemical analysis and SEM/TEM studies are recommended to analyze the real interaction of paste on tooth surfaces when using different types of glass ionomer cements.

## Conclusion

Within the limitations of this study and regarding the results, it can be concluded that CPP-ACP paste pretreatment do not affect the microleakage of self-cured or nanofilled glass ionomer cements in enamel or dentin margin, except in dentinal margins of resin-modified glass ionomer (Fuji II LC) when cavity conditioner is applied before surface treatment.
